# In silico analysis to identify vaccine candidates common to multiple serotypes of *Shigella* and evaluation of their immunogenicity

**DOI:** 10.1371/journal.pone.0180505

**Published:** 2017-08-02

**Authors:** Sapna Pahil, Neelam Taneja, Hifzur Rahman Ansari, G. P. S. Raghava

**Affiliations:** 1 Department of Medical Microbiology, Postgraduate Institute of Medical Education and Research, Chandigarh, India; 2 Bioinformatics Centre, Institute of Microbial Technology, Chandigarh, India; International Centre for Diarrhoeal Disease Research Bangladesh (icddr,b), BANGLADESH

## Abstract

Shigellosis or bacillary dysentery is an important cause of diarrhea, with the majority of the cases occurring in developing countries. Considering the high disease burden, increasing antibiotic resistance, serotype-specific immunity and the post-infectious sequelae associated with shigellosis, there is a pressing need of an effective vaccine against multiple serotypes of the pathogen. In the present study, we used bio-informatics approach to identify antigens shared among multiple serotypes of *Shigella* spp. This approach led to the identification of many immunogenic peptides. The five most promising peptides based on MHC binding efficiency were a putative lipoprotein (EL PGI I), a putative heat shock protein (EL PGI II), Spa32 (EL PGI III), IcsB (EL PGI IV) and a hypothetical protein (EL PGI V). These peptides were synthesized and the immunogenicity was evaluated in BALB/c mice by ELISA and cytokine assays. The putative heat shock protein (HSP) and the hypothetical protein elicited good humoral response, whereas putative lipoprotein, Spa32 and IcsB elicited good T-cell response as revealed by increased IFN-γ and TNF-α cytokine levels. The patient sera from confirmed cases of shigellosis were also evaluated for the presence of peptide specific antibodies with significant IgG and IgA antibodies against the HSP and the hypothetical protein, bestowing them as potential future vaccine candidates. The antigens reported in this study are novel and have not been tested as vaccine candidates against *Shigella*. This study offers time and cost-effective way of identifying unprecedented immunogenic antigens to be used as potential vaccine candidates. Moreover, this approach should easily be extendable to find new potential vaccine candidates for other pathogenic bacteria.

## Introduction

Shigellosis is a highly infectious acute gastroenteritis and as few as 10 to 100 bacteria are capable of causing the disease [1]. Around 90 million cases of the severe disease occur each year with 108,000 deaths, most of which occur in the developing countries. Children under 5 years of age are mainly affected [2,3]. The recent diagnosis studies using quantitative polymerase chain reaction (qPCR) have indicated that the traditional culture-based methods have under-estimated the global burden of shigellosis [4,5]. The disease also affects travelers to developing countries, military personnel, refugees and the institutionalized persons [2,6,7]. In the Indian subcontinent, the disease occurs endemically as well as many outbreaks are reported from time to time [8–13].

*Shigella* has four major serogroups and more than 50 serotypes exist based on antigenic differences in O-antigen of outer membrane LPS (lipo-polysaccharide). *Shigella dysenteriae* serogroup (group A, 17 serotypes) includes *S*. *dysenteriae* type1, which causes epidemic and pandemic disease. *Shigella flexneri* (group B, 14 serotypes) is endemic to developing nations and accounts for most infections worldwide. *Shigella sonnei* (group C, one serotype) mainly causes outbreaks in the developed countries and *Shigella boydii* (group D, 20 serotypes) is endemic to the Indian subcontinent [14].

The treatment of shigellosis is complicated by the therapeutic challenge posed by the emerging multidrug resistance against *Shigella* [15–20]. Availability of more precise data on disease burden and antibiotic resistance has further amplified the need for a safe and effective vaccine against *Shigella* [13,15,18,21,22]. The extensive research into *Shigella* pathogenesis as well as the immune response studies of natural infection cases or experimental human volunteers have failed to establish a definite correlate of protection, thereby significantly hampering the vaccine development [21]. *Shigella* invades the colonic epithelial cells with a surface exposed needle like structure called as Type III secretion system (TTSS), formed by Mxi and Spa proteins. The secretory proteins of TTSS, together with some effector proteins mediate invasion and intercellular spread of *Shigella* [23]. Many studies have reported antibodies from infected humans’ sera against these surface exposed or secreted antigens (LPS, Ipa proteins, IcsA), leading to their exploitation in various vaccine strategies such as live-attenuated, inactivated whole cell, sub-cellular and purified sub-unit vaccines [24,25]. Many vaccines based on O-antigen have been developed in the past but faced protection issues in children under age of 2 years. For example, SmD (streptomycin-dependent) *Shigella* vaccine, which was tested on a large scale, reverted to streptomycin independence leading to difficulties in the process control [26]. The protection levels of some vaccines differed in ethinically different field trials [27] and the live attenuated vaccines induced unexpectable reactogenicity with dose escalation [28]. Moreover, O-antigens generated serotype-specific immunity, leading to poor cross-reactivity among different serotypes of *Shigella* [29,30]. The live attenuated vaccines were either over-attenuated (poor immune response) or had unacceptable reactogenicity [31]. A promising vaccine strategy underway is Invaplex vaccine that combines LPS with different Ipa proteins (IpaB, IpaC or IpaD). However, such combinatorial approaches need to be tested for formulations, immunogenicity and adverse reactions [32–34]. The serotype-specific immunity requiring coverage of more than 50 serotypes is a major impediment to vaccine development against *Shigella* [21,30,35,36]. A ‘global’ *Shigella* vaccine must provide protection against *S*. *dysenteriae* type1, *S*. *sonnei* and *S*. *flexneri* 2a, as these contribute to the majority of cases of shigellosis [37]. The above mentioned challenges demonstrate a need to explore novel vaccine candidates [38].

The availability of whole genome and proteome sequences of many pathogenic microorganisms has accelerated the identification of bacterial/viral vaccine candidates [39–42]. The first successful vaccine using above strategy was developed against Meningococcus B strains [43]. Proteins can be screened with ease for B- and T-cell epitopes by various prediction algorithms and such predicted epitopes can be tested for their efficacy as peptide vaccines [44,45]. Many peptides have been tested in preclinical and clinical trials for a large number of pathogens such as *P*. *gingivalis*, Semliki forest virus, Foot and mouth disease virus, Human papilloma virus and even in the cancer patients [46–54].

In the present study, we used many widely referenced in-silico tools for the prediction of immunogenic peptides, conserved across different serotypes to overcome serotype-specific immunity and then tested them *in-vivo* in BALB/c mice. The whole proteomes (available at the start of the study in 2009) of three major serotypes of *Shigella*; *S*. *dysenteriae* type 1, *S*. *flexneri 2a* and *S*. *sonnei* were retrieved from publicly available database of NCBI and a local database of proteins was created. The proteins common to the above-mentioned serotypes were identified by performing protein-protein BLAST. From the shared proteins; surface, outer-membrane or secreted proteins were selected utilizing various localization prediction algorithms. The selected proteins were then screened for B- and T-cell epitopes as well as MHC binding efficiency (IC50 value) by various algorithms. The identified immunogenic epitopes were commercially synthesized as peptides and their immunogenicity (both humoral and cell mediated response) was checked in BALB/c mice. We also investigated the effect of different routes of immunization on immune response generation by inoculating the same peptide by three routes. The intranasal and subcutaneous routes induced comparable immune responses.

The epidemiological surveillance of a cohort of children in an endemic area [55] and human volunteer re-challenge studies [56,57] have depicted that serotype-specific homologous protection exists against natural *Shigella* infection. Therefore, we also tested the presence of naturally occurring antibodies against the identified antigens in shigellosis patient sera. The predicted peptides evoked the immune response not only in BALB/c mice but antibodies against the same were also found in patients’ sera. Our study has led to the identification of novel antigens against *Shigella*, which are conserved and cross-protective and can serve as potential vaccine candidates. It also highlights the importance of bioinformatics as an alternative strategy to discover new antigens.

## Materials and methods

### 2.1 Retrieving protein sequences and BLAST (Basic Local Alignment Search Tool)

The proteome files of completely sequenced strains of three serotypes of *Shigella*; *S*. *dysenteriae* type1 (sd197), *S*. *flexneri 2a* (str. 301 and str. 2457T) and *S*. *sonnei* (ss046) were retrieved in FASTA format from publicly available database at NCBI (http://www.ncbi.nlm.nih.gov) and a local database of all these proteins was created. The retrieved sequences were subjected to all versus all Protein-BLAST to obtain proteins common in three serotypes. The BLAST was run with an e-value threshold of 1×e-^4^ and filter parameter of >75% similarity.

### 2.2 Protein localization prediction

Sub-cellular localization predictions of the selected common proteins were performed to identify surface, outer-membrane or secreted proteins using the following localization prediction softwares: PSORTb (http://www.psort.org/psortb/index.html) [58], Cello (http://cello.life.nctu.edu.tw/) [59], SubLoc (http://www.bioinfo.tsinghua.edu.cn/subloc/) [60] and Pslpred (http://www.imtech.res.in/raghava/submit.html) [61]. For localization predictions, the protein sequences were taken in FASTA format, the organism type was chosen as ‘Bacteria’, Gram stain was selected as ‘Negative’ and the output format was selected as normal in the required field. These softwares have various support vector machines (SVMs) classifiers, trained to identify different locations based on amino acid composition or physic-chemical properties of proteins. All nuclear, periplasmic or cytoplasmic proteins were filtered out.

### 2.3 B-cell and T-cell epitopes prediction

The selected proteins were screened for identification of B-cell epitopes using prediction softwares; Bepipred (http://www.cbs.dtu.dk/services/BepiPred/) [62] and Bcepred (http://www.imtech.res.in/raghava/bcepred/) [63]. The Bepipred is based on hidden Markov model and propensity scale, whereas Bcepred uses physico-chemical properties such as flexibility, hydrophilicity, polarity etc. The score threshold for epitope assignment was kept at default value of 0.35 for Bepipred and at 2.38 for Bcepred. The residues with scores above the threshold are predicted as B-cell epitopes. T-cell epitope predictions were carried out by nHLAPRED (http://www.imtech.res.in/raghava/nhlapred/) [64], IEDB (Immune Epitope Database) (http://tools.iedb.org/mhci/) [65] for MHC class-I binders and by PROPRED (http://www.imtech.res.in/raghava/propred/) [66] and IEDB (http://tools.iedb.org/mhcii/) [65] for MHC Class-II binders. These softwares are based on either artificial neural network (ANN) or stabilized matrix method (SMM) except Propred, which uses quantitative matrices for prediction. The IEDB is the largest immune-epitope database available. For predicting T-cell epitopes, ‘IEDB Recommended’ (default prediction method) is selected, which uses the best possible prediction for a given MHC molecule. Finally, an in-house Perl-script was used to select common epitopes by B- and T-cell prediction algorithms. The epitopes were also subjected to IC50 value (MHC binding affinity) prediction by IEDB, by using stabilized matrix method (SMM), which determines their affinity to the specific MHC alleles.

### 2.4 Peptides selection and synthesis

Total five peptides were selected from predicted antigens (n = 48), based on two-fold criteria. First, the peptides, which had B- and T-cell epitopes common by multiple softwares (four or more), attributing them increased prediction accuracy and second, peptides having high MHC binding score. The peptides selected were a putative lipoprotein (EL PGI I), a putative heat shock protein (EL PGI II), Spa32 (EL PGI III), IcsB (EL PGI IV) and a hypothetical protein (EL PGI V). These were commercially synthesized from USV (United States Vitamins Ltd., Mumbai, India) and were of >95 percentage purity, tested by HPLC (high performance liquid chromatography).

### 2.5 Animals used in study

Five to six-weeks old, female BALB/c mice were taken and kept at Institute’s animal facility. All experiments were performed after ethical clearance and according to the guidelines of the Animal Ethics Committee of the Postgraduate Institute of Medical Education and Research (PGIMER), Chandigarh, India. The ketamine-xylazine cocktail (1ml of ketamine containing 100 mg/ml and 0.5 ml of xylazine containing 20 mg/ml and 8.5 ml saline) of 0.1 ml/10g was used for anesthesia to minimize the pain of injection and thrombophobe cream was applied on the site of injection. Feed and water were provided round the clock.

### 2.6 Immunization of mice

For each peptide to be tested, the BALB/c mice were divided into three sub-groups, one test and two control groups, each group comprised of six mice. The mice in each group were injected either subcutaneously (s.c), intraperitoneally (i.p) or intranasally (i.n) for all the peptides tested. On day 0, mice were injected with 100 μl dose, consisting of 50 μg of each peptide emulsified in equal volume of complete Freund’s adjuvant (CFA), followed by two booster doses of the peptide emulsified in incomplete Freund’s adjuvant (IFA) on days 14 and 28. Two control groups were injected with either PBS alone or PBS with adjuvant similar to test groups. We also immunized the mice with a mixture of all five peptides to compare the immune response generated by individual peptide versus the mixture. The mixture was prepared by adding equal amounts of each peptide (1/5^th^ of the individual dose) emulsified in equal volume of CFA for primary dose and IFA for booster doses. After three weeks of the last dose, blood was collected from the mice by retro-orbital plexus. The serum was separated and stored in two aliquots at -80°C until further use.

### 2.7 Human sera collection

The blood samples were collected from informed human volunteers after written consent following standard laboratory procedure. The samples were taken from the convalescent patients (n = 15) suffering from shigellosis, confirmed by stool culture and also from age matched healthy controls (n = 10) with zero episodes of diarrhea in past six months. The stool cultures confirmed the presence of following serogroups; *Shigella flexneri* (73.33%), *S*. *sonnei* (6.66%), *S*. *boydii* (6.66%) and NAG (non-agglutinable *Shigella*) (13.33%). These sera were checked for the presence of naturally occurring peptide-specific IgG and IgA antibodies against the same peptides that were tested in mice. We also investigated the cytokine response for both Th1 and Th2 type in these sera by flow cytometry to compare the elevation of cytokines in the immunized mice versus humans during natural course of infection.

### 2.8 Enzyme-linked immunosorbent assay (ELISA)

The antibody detection (IgG and IgA) in mice sera was done by micro ELISA technique. Checkerboard titration method was used to determine optimum antigen concentration and serum dilutions for ELISA. Following antigen concentrations: 2.5, 5 and 10 μg/well and serum dilutions starting from 1:10 and up to 1:3200 were tested. The cut-off value was identified as the highest dilution at which absorbance value of a sample differed significantly from a healthy control. For each set of ELISA, the cut-off OD (optical density) was calculated by taking mean of the OD values of five negative controls and adding two standard deviation (+2 SD) to it. The samples with OD values equal to or more than cut-off OD values were taken as positive. Finally, antigen concentration of 10 μg/well, serum dilutions of 1:40, 1:80, and 1:160 for IgG and 1:5, 1:10 for IgA were used. Similarly, shigellosis patients’ sera were also checked for the peptide-specific antibodies by ELISA. ELISA was performed as described previously [67].

### 2.9 Cytokine assay

Cytokine analysis was done by flow cytometry using the mouse and human Th1 and Th2 cytokine kits (BD Biosciences, USA). Six cytokines; IL-2, IL-4, IL-1β, IL-10, IFN-γ and TNF-α were tested in the serum samples of the test and control groups of mice. Similarly, we looked for these cytokines in shigellosis patients’ sera versus healthy controls. The assays were performed according to manufacturer’s instructions.

### 2.10 Statistical analysis

The experimental data were analyzed by Graph-Pad Prism version 3 (free trial version) and expressed as mean± 2SD. Comparisons between individual data points were made using Mann-Whitney U test and the p-value of <0.05 was considered as statistically significant. The antibody response in the humans was analyzed by independent t-test and p-value of <0.05 was considered as statistically significant.

## Results

### 3.1 Protein database creation and BLAST

A local database of proteins was created after merging protein files of all the retrieved strains of the completely sequenced serotypes of *S*. *dysenteriae* type1 (sd197), *S*. *flexneri* 2a (str. 301, str. 2457T) and *S*. *sonnei* (ss046). Table 1 lists the number of proteins from each strain that were used in this analysis. Each protein query showing hits with all the four strains above BLAST threshold was considered and rests were filtered out. The proteins identified as common after BLAST were further processed for the prediction of cellular localization.

**Table 1:** Different serotypes and strains of *Shigella* used in local database creation.

S. No.	Serotype (strain)	Bio-project	Chromosomes	Plasmids	Genes	Proteins
1.	*S*. *flexneri* 2a (str. 301)	PRJNA62907, PRJNA301	1	1	4,828	4,438
2.	*S*. *flexneri* 2a (str. 2457T)	PRJNA57991 PRJNA408	1	-	4,570	4,060
3.	*S*. *dysenteriae* (Sd197)	PRJNA58213 PRJNA13145	1	2	4,892	4,501
4.	*S*. *sonnei* (Ss046)	PRJNA58217 PRJNA13151	1	4	4,804	4,470

The Table 1 lists the completely sequenced serotypes (till the year 2009) and their strains used in the study. The numbers of chromosomes and plasmids for each strain and the total proteins encoded by them, which were used in local database creation, are also indicated.

### 3.2 Protein localization prediction

The prediction softwares display the results as scores on the scale of 0–10 for each of the five sites (cytoplasmic, cytoplasmic-membrane, periplasmic, extracellular and outer-membrane). For a particular protein, if three out of four softwares predicted similar localization, we would consider that as positive. After localization predictions, 250 proteins were identified as outer-membrane/surface or secreted proteins. These were further processed for epitopes prediction. These 250 proteins with their identifications and localizations are provided in the excel file (attached as supplementary material, S1 Fig).

### 3.3 Selection and synthesis of B- and T-cell epitopes

The selected outer-membrane or secreted proteins yielded around 39,000 peptides as common epitopes (containing both B- and T-cell epitopes, but majority of them consisted of very few amino acids. Around 48 peptides that were ≥9 amino acids long (a consideration to elicit B- or T-cell response) were identified. Due to financial constraint, only 5 were selected for immunogenicity evaluation in BALB/c mice on the basis of high MHC binding affinity score (IC50 value ranges 674–5199) supporting their prediction as antigens. The commercially synthesized peptides sequences and their respective gene bank accession numbers are shown in Table 2.

**Table 2:** The peptides identified by in-silico analysis and used for in-vivo experiments.

S No.	Protein Name	Gene Bank Accession Number	Peptide Sequence	Localization	Function/ Comments
1	Putative lipoprotein (EL PGI I)	|NP_837342.1| (WP_000101178.1)	AKKAASKSMTKSKTA	Outer membrane	Mostly bacterial, absent in primates, function is not clear.
2	Putative heat shock protein (EL PGI II)	|YP_403917.1|	DSGNQVASKSSKYGK	Outer membrane	Proteolysis and metallo-endopeptidase activity
3	Spa32 (EL PGI III)	|YP_313368.1| (WP_001162455.1)	NADKNFERSHTSSVNPDNL	Secreted	Member of type III (virulence related) secretory pathway
4	IcsB (EL PGI IV)	|YP_406171.1|	LLLFLKTLASEKSAESAFAA	Secreted	Virulence protein
5	Hypothetical Protein (EL PGI V)	|NP_837127.1| (WP_000163771.1)	YGVSRKESARSGLRGYN	Outer membrane	mltA interacting protein, mostly bacterial, absent in primates.

Table 2 shows the five selected proteins after in-silico analysis. The proteins are shared among multiple serotypes of *Shigella* and are either outer-membrane or secreted in nature. The immunogenic peptide sequences identified by multiple B- and T-cell epitope prediction softwares are shown. These were synthesized and used for in-vivo immunization experiments. Functional analysis of selected proteins was done by the InterProScan software.

### 3.4 Antibody response in mice

**Fig 1:**
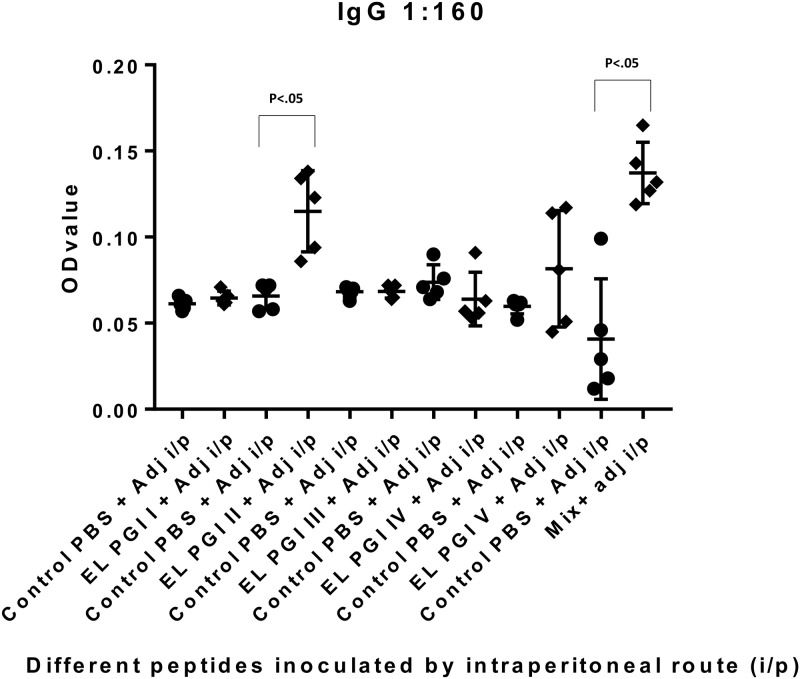
IgG antibody response in BALB/c mice against all five peptides and their mixture inoculated by intraperitoneal route. Antibody titers in sera of mice primed intraperitoneally with peptide + CFA and boosted twice with peptide + IFA are shown. The X-axis depicts all five peptides, their mixture inoculated with adjuvant and respective control (PBS+ adjuvant) for each peptide. OD values of peptides vs. controls are depicted on Y- axis. Absorbance was measured at 450nm. Data collected from 6 mice per group (2 separate experiments) are expressed as geometric mean concentration (± 2SD) at serum dilution of 1:160. Statistical significance was determined by Mann-Whitney U test and the p-value of < 0.05 was taken as significant.

**Fig 2:**
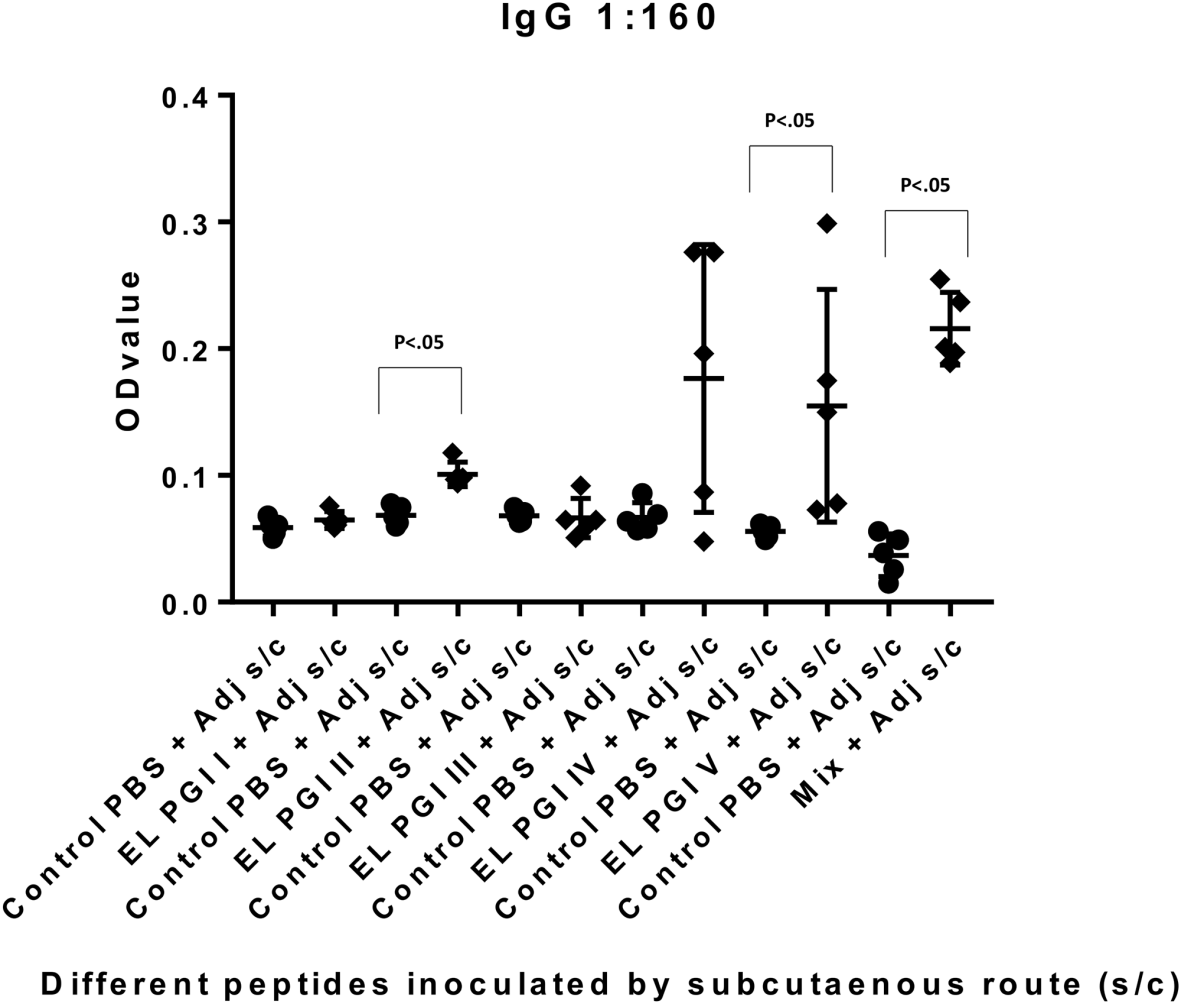
IgG antibody response in BALB/c mice against all five peptides and their mixture inoculated by subcutaneous route. Antibody titers in sera of mice primed subcutaneously with peptide + CFA and boosted twice with peptide + IFA are shown. The X-axis depicts all five peptides, their mixture inoculated with adjuvant and respective control (PBS+ adjuvant) for each peptide. OD values of peptides vs. controls are depicted on Y- axis. Absorbance was measured at 450nm. Data collected from 6 mice per group (2 separate experiments) are expressed as geometric mean concentration (± 2SD) at serum dilution of 1:160. Statistical significance was determined by Mann-Whitney U test and the p-value of < 0.05 was taken as significant.

**Fig 3:**
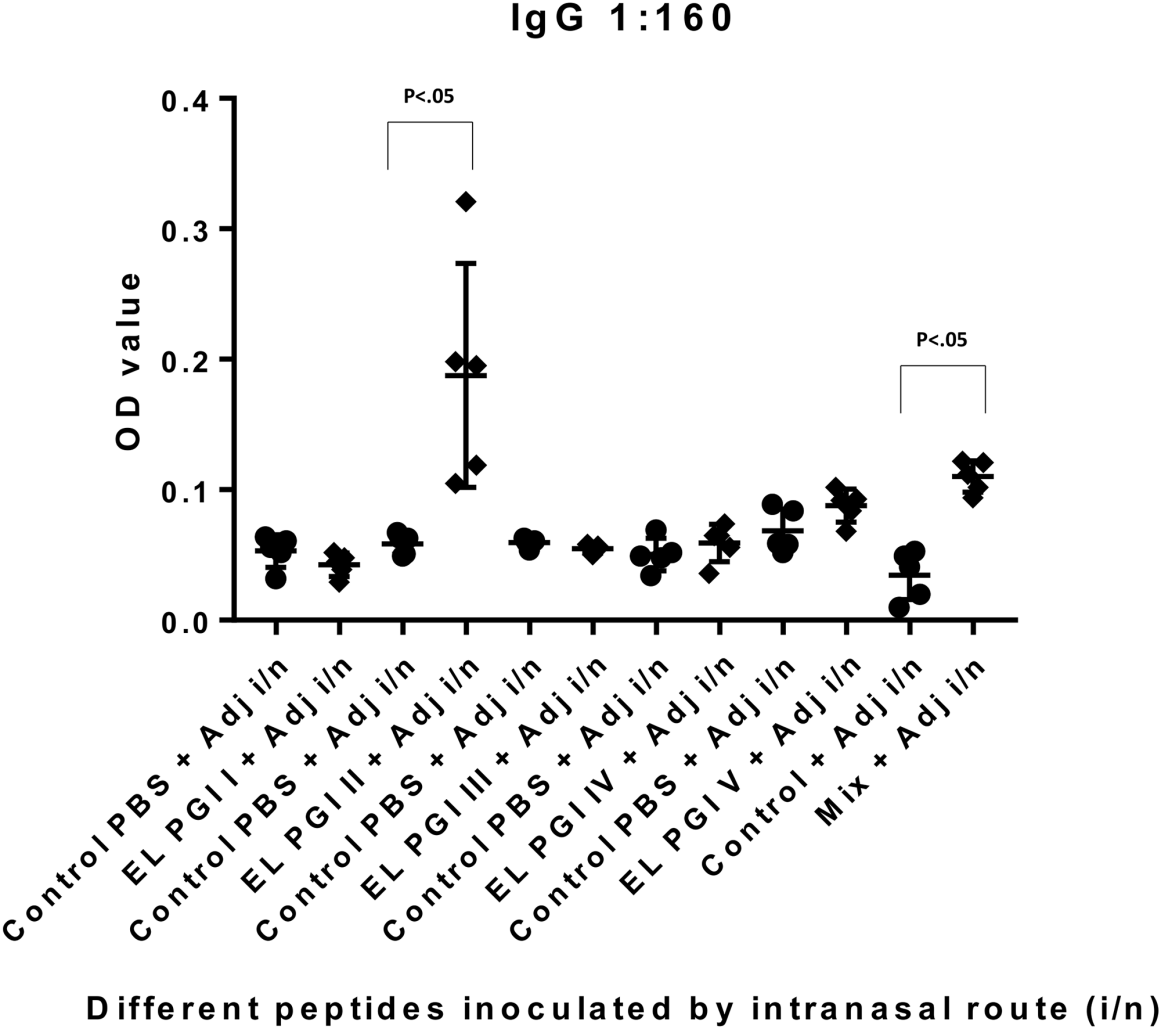
IgG antibody response in BALB/c mice against all five peptides and their mixture inoculated by intranasal route. Antibody titers in sera of mice primed intranasally with peptide + CFA and boosted twice with peptide + IFA are shown. The X-axis depicts all five peptides, their mixture inoculated with adjuvant and respective control (PBS+ adjuvant) for each peptide. OD values of peptides vs. controls are depicted on Y- axis. Absorbance was measured at 450nm. Data collected from 6 mice per group (2 separate experiments) are expressed as geometric mean concentration (± 2SD) at serum dilution of 1:160. Statistical significance was determined by Mann-Whitney U test and the p-value of < 0.05 was taken as significant.

**Fig 4:**
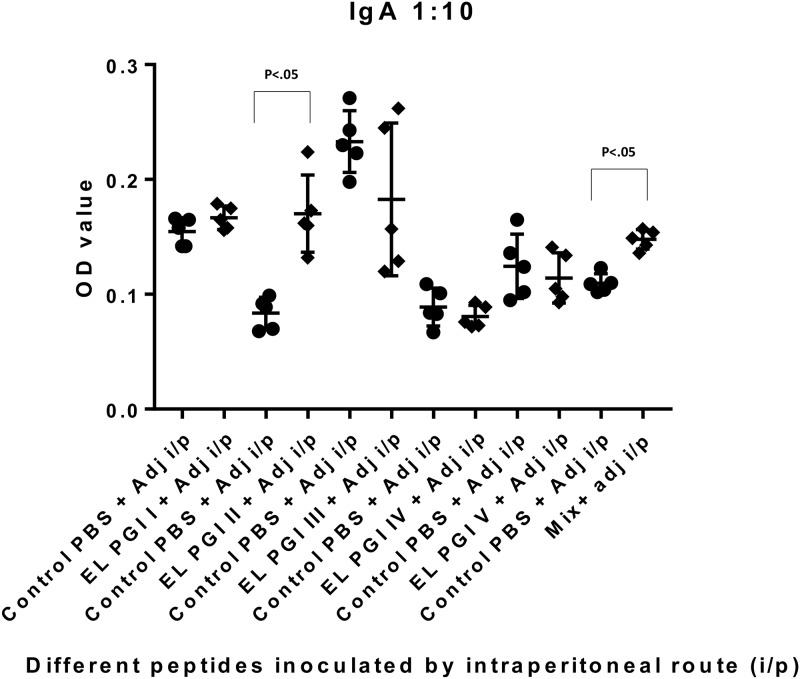
IgA antibody response in BALB/c mice against all five peptides and their mixture inoculated by intraperitoneal route. Antibody titers in sera of mice primed intraperitoneally with peptide + CFA and boosted twice with peptide + IFA are shown. The X-axis depicts all five peptides, their mixture inoculated with adjuvant and respective control (PBS+ adjuvant) for each peptide. OD values of peptides vs. controls are depicted on Y- axis. Absorbance was measured at 450nm. Data collected from 6 mice per group (2 separate experiments) are expressed as geometric mean concentration (± 2SD) at serum dilution of 1:160. Statistical significance was determined by Mann-Whitney U test and the p-value of < 0.05 was taken as significant.

**Fig 5:**
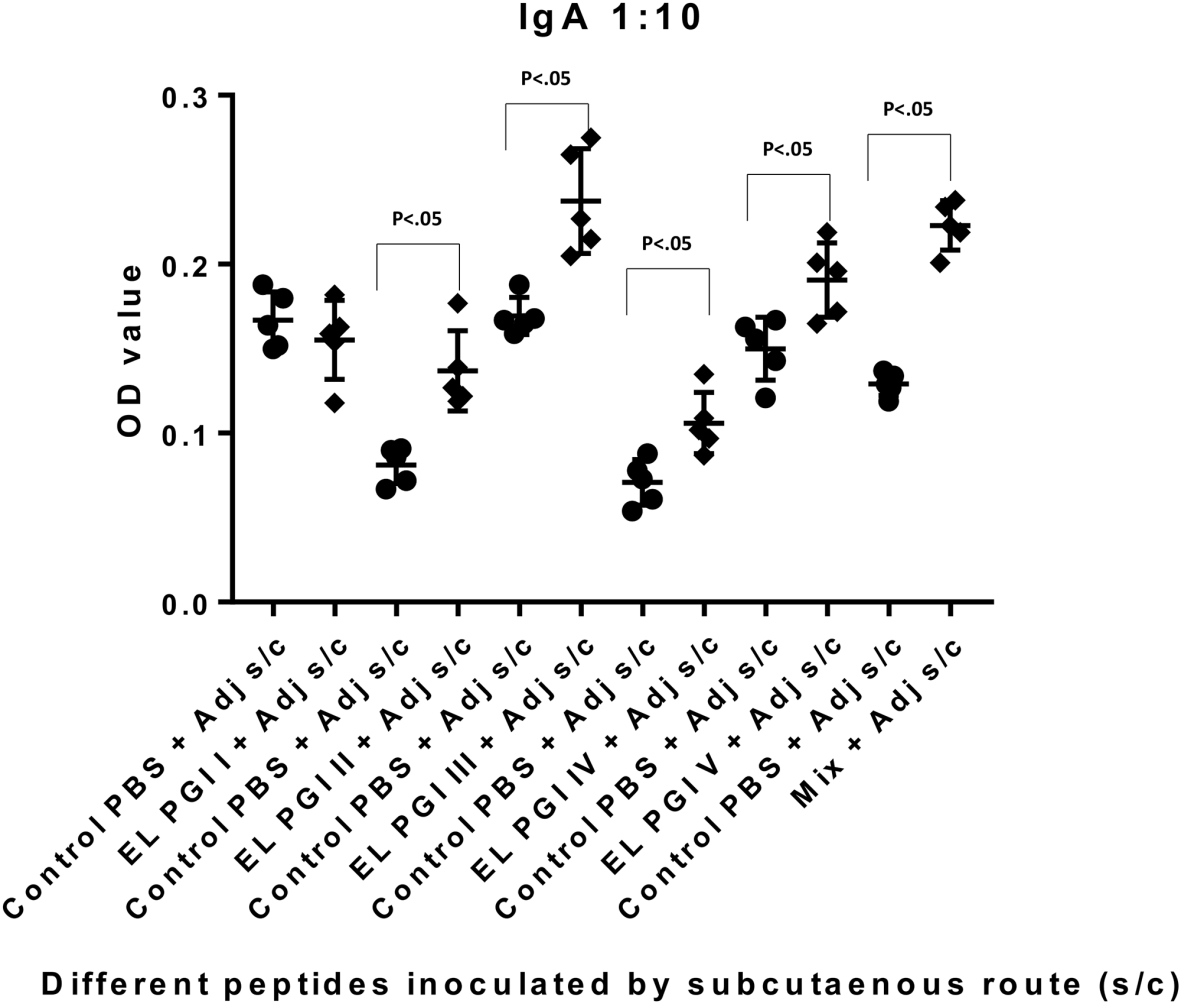
IgA antibody response in BALB/c mice against all five peptides and their mixture inoculated by subcutaneous route. Antibody titers in sera of mice primed subcutaneously with peptide + CFA and boosted twice with peptide + IFA are shown. The X-axis depicts all five peptides, their mixture inoculated with adjuvant and respective control (PBS+ adjuvant) for each peptide. OD values of peptides vs. controls are depicted on Y- axis. Absorbance was measured at 450nm. Data collected from 6 mice per group (2 separate experiments) are expressed as geometric mean concentration (± 2SD) at serum dilution of 1:160. Statistical significance was determined by Mann-Whitney U test and the p-value of < 0.05 was taken as significant.

**Fig 6:**
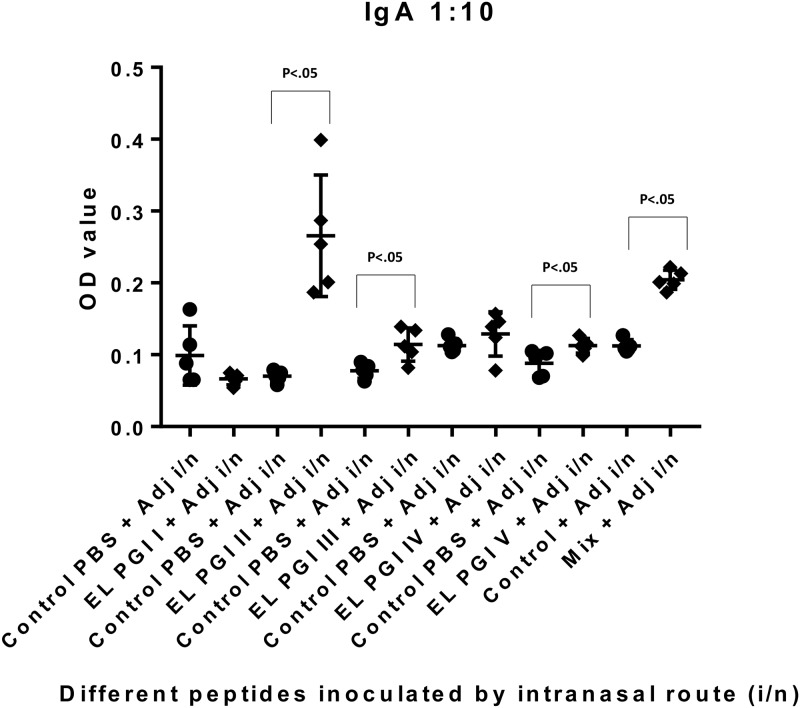
IgA antibody response in BALB/c mice against all five peptides and their mixture inoculated by intranasal route. Antibody titers in sera of mice primed intranasally with peptide + CFA and boosted twice with peptide + IFA are shown. The X-axis depicts all five peptides, their mixture inoculated with adjuvant and respective control (PBS+ adjuvant) for each peptide. OD values of peptides vs. controls are depicted on Y- axis. Absorbance was measured at 450nm. Data collected from 6 mice per group (2 separate experiments) are expressed as geometric mean concentration (± 2SD) at serum dilution of 1:160. Statistical significance was determined by Mann-Whitney U test and the p-value of < 0.05 was taken as significant.

Indirect ELISA was used to measure antigen specific IgG and IgA antibody response. Antibody titers of both IgG and IgA were insignificant (p>0.05) for putative lipoprotein (EL PGI I), for all the three routes of inoculation (Figs 1–6). Putative heat shock protein (EL PGI II) exhibited significant titers (p<0.05) of both IgG and IgA antibodies by all the three routes (Figs 1–6). Spa32 (EL PGI III) did not elicit significant IgG antibody titers by any of the routes (p>0.05, Figs 1–3) but elicited significant IgA response (p<0.05) by both subcutaneous and intranasal route (Figs 5 and 6). IcsB (EL PGI IV) elicited significant IgA antibody titers by subcutaneous route alone (Fig 5), whereas IgG titers were insignificant for any of the three routes of immunization (Figs 1–3). Hypothetical protein (EL PGI V) elicited significant IgG antibody titers by subcutaneous route only (Fig 2), whereas IgA titers were significant by both subcutaneous and intranasal routes (Figs 5 and 6). However, the cocktail of all the five peptides elicited significant (p<0.05) titers of both IgG and IgA antibodies by all the three different routes as compared to the controls (Figs 1–6).

### 3.5 Peptide specific antibodies in shigellosis patients’ sera

Serum samples of shigellosis patients (n = 15) and age matched healthy controls (n = 10) were tested by ELISA for the presence of peptide-specific IgG and IgA antibodies. The antibody titers of healthy controls were compared with patient sera by independent t-test as shown in Table 3. Both IgG and IgA antibodies against the putative heat shock protein (EL PGI II) and the hypothetical protein (EL PGI V) were significantly present (p<0.05) in the patient sera as compared to healthy controls. The IgG titers against putative lipoprotein (EL PGI I) and IcsB (EL PGI IV) were also significant (p<0.05) but the IgA antibody response was insignificant. The antibody response for both IgG and IgA was insignificant (p>0.05) for Spa32 (EL PGI III).

**Table 3:** IgG and IgA antibody response against selected peptides in shigellosis patients’ versus control sera.

Peptides		Group	N	Mean	Std. Deviation	Std. Error Mean	P-value
EL PGI I	IgG	Controls	10	.04260	.016234	.004192	.029*
		Patients	15	.06520	.034528	.008915	
	IgA	Controls	10	.02107	.003327	.000859	.162
		Patients	15	.02280	.003278	.000846	
EL PGI II	IgG	Controls	10	.04880	.009897	.003130	<.001*
		Patients	15	.22740	.034830	.008993	
	IgA	Controls	10	.02120	.002700	.000854	<.001*
		Patients	15	.11420	.036511	.009427	
EL PGI III	IgG	Controls	10	.04690	.008144	.002575	.145
		Patients	15	.05520	.016010	.004134	
	IgA	Controls	10	.02520	.005266	.001665	.063
		Patients	15	.03853	.021027	.005429	
EL PGI IV	IgG	Controls	10	.04930	.008233	.002604	.011*
		Patients	15	.07840	.032151	.008301	
	IgA	Controls	10	.02910	.009339	.002953	.131
		Patients	15	.03760	.015329	.003958	
EL PGI V	IgG	Controls	10	.04210	.006790	.002147	<.001*
		Patients	15	.23487	.056600	.014614	
	IgA	Controls	10	.02800	.007944	.002512	<.001*
		Patients	15	.10080	.035944	.009294	

Table 3 depicts the presence of IgG and IgA antibodies in human sera against the selected peptides. The antibody titers were measured by ELISA. The comparison of antibody titers between patients’ vs. control sera was done by Independent t-test to calculate statistical significance and p-value was taken significant at the .05 level.

* the p-value is significant.

### 3.6 Cytokine levels in mice sera

**Fig 7:**
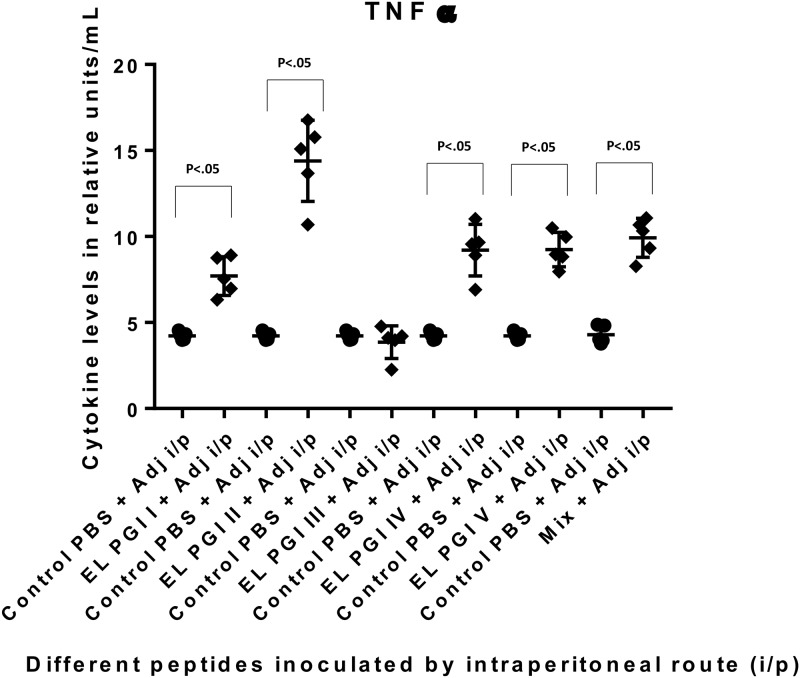
TNF-α levels in BALB/c mice against all peptides and their mixture inoculated by intraperitoneal route. On the X-axis are shown all five peptides with the adjuvant, their mixture and controls (PBS+ Adjuvant) inoculated intraperitoneally. Mice were primed with peptide + CFA and two booster doses at two weeks interval each were given with peptide + IFA. TNF-α levels in pg/ml in the undiluted sera are shown on Y-axis. Data collected from 6 mice per group (2 separate experiments) are expressed as geometric mean concentration (± 2SD). Statistical significance was determined by Mann-Whitney U test and the p-value of < 0.05 was taken as significant.

**Fig 8:**
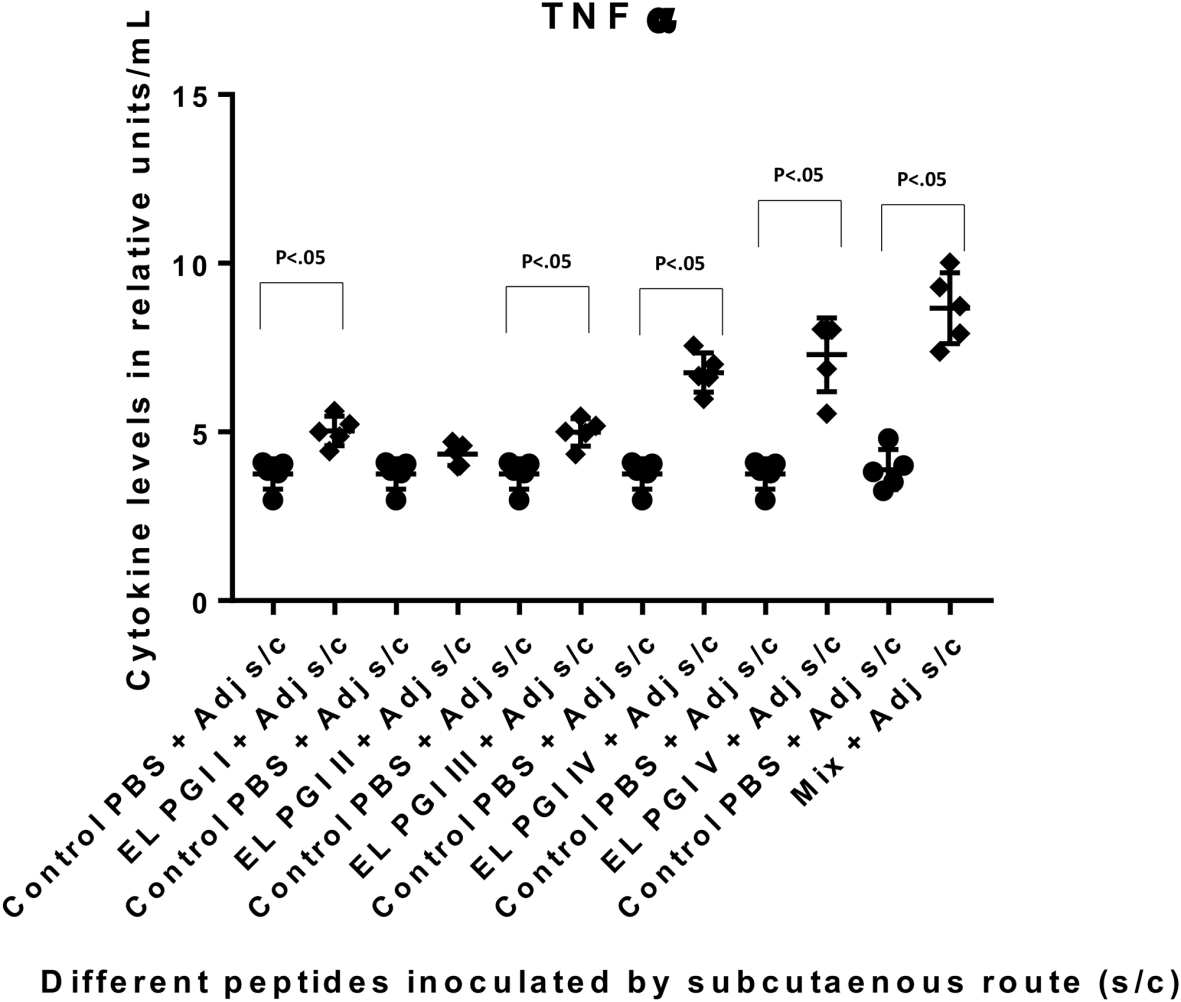
TNF-α levels in BALB/c mice against all peptides and their mixture inoculated by subcutaneous route. On the X-axis are shown all five peptides with the adjuvant, their mixture and controls (PBS + Adjuvant) inoculated subcutaneously. Mice were primed with peptide + CFA and two booster doses at two weeks interval each were given with peptide + IFA. TNF-α levels in pg/ml in the undiluted sera are shown on Y-axis. Data collected from 6 mice per group (2 separate experiments) are expressed as geometric mean concentration (± 2SD). Statistical significance was determined by Mann-Whitney U test and the p-value of < 0.05 was taken as significant.

**Fig 9:**
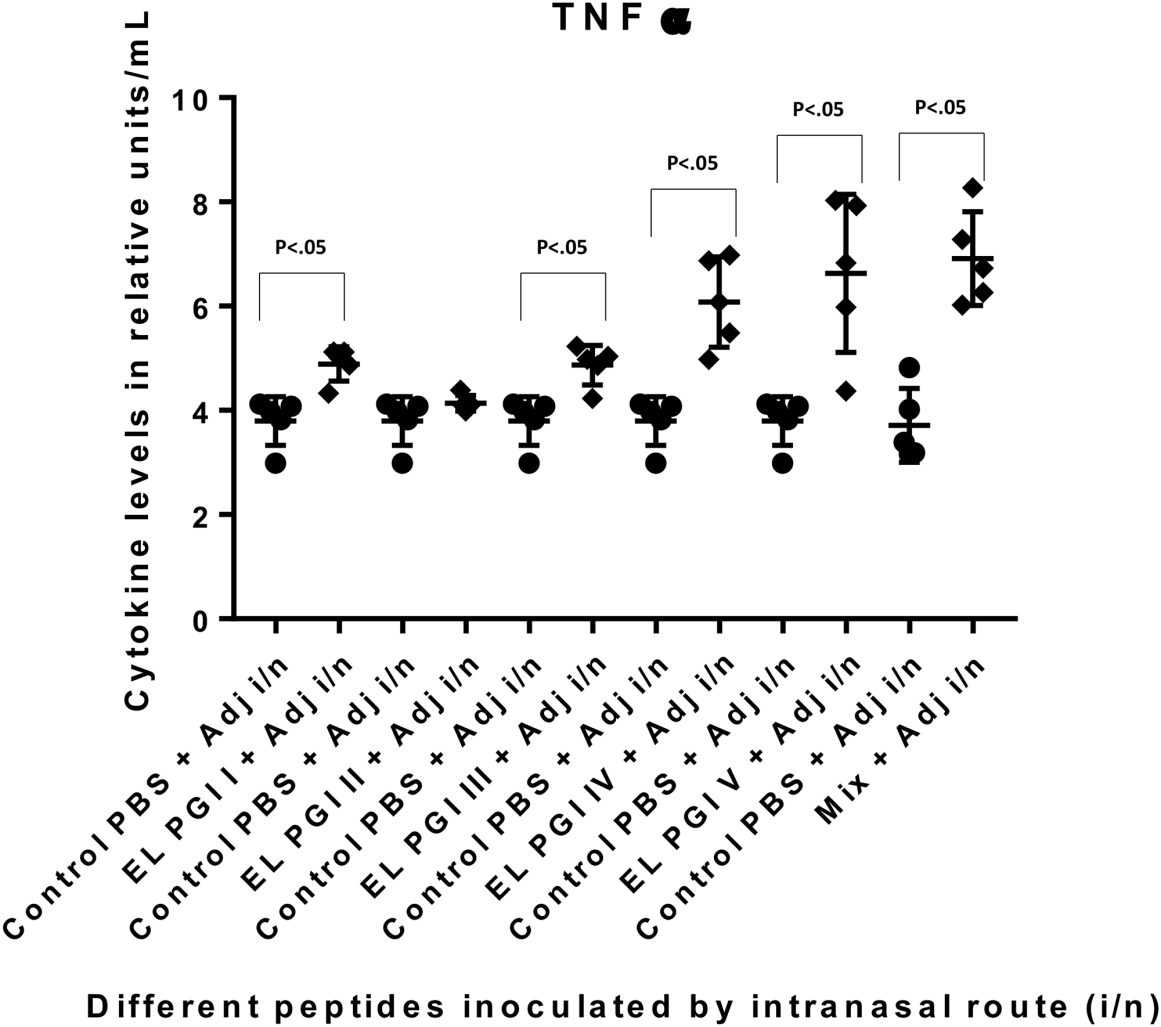
TNF-α levels in BALB/c mice against all peptides and their mixture inoculated by intranasal route. On the X-axis are shown all five peptides with the adjuvant, their mixture and controls (PBS + Adjuvant) inoculated intranasally. Mice were primed with peptide + CFA and two booster doses at two weeks interval each were given with peptide + IFA. TNF-α levels in pg/ml in the undiluted sera are shown on Y-axis. Data collected from 6 mice per group (2 separate experiments) are expressed as geometric mean concentration (± 2SD). Statistical significance was determined by Mann-Whitney U test and the p-value of < 0.05 was taken as significant.

Cytokine levels (Th1 and Th2) were measured by capture bead assay (CBA) using a flow cytometer (BD FACS CANTO). Six cytokines; IL-2, IL-4, IL-1β, IL-10, IFN-γ and TNF-α were tested in the sera of the test and control groups. There was no significant increase in the levels of IL-2, IL-4, IL-1β and IL-10 by any of the three routes of immunization. TNF-α was significantly elevated (p<0.05) against the putative lipoprotein (EL PGI I), IcsB (EL PGI IV) and the hypothetical protein (EL PGI V) by all routes (Figs 7–9). However, the putative heat shock protein (EL PGI II) elicited significant levels of TNF-α (p<0.05) only by intraperitoneal route (Fig 7). Spa32 (EL PGI III) elicited TNF-α significantly by subcutaneous and intranasal routes (Figs 8 and 9). The cocktail of all the five peptides showed significant levels of TNF-α (p<0.05) by all routes (Figs 7–9).

**Fig 10:**
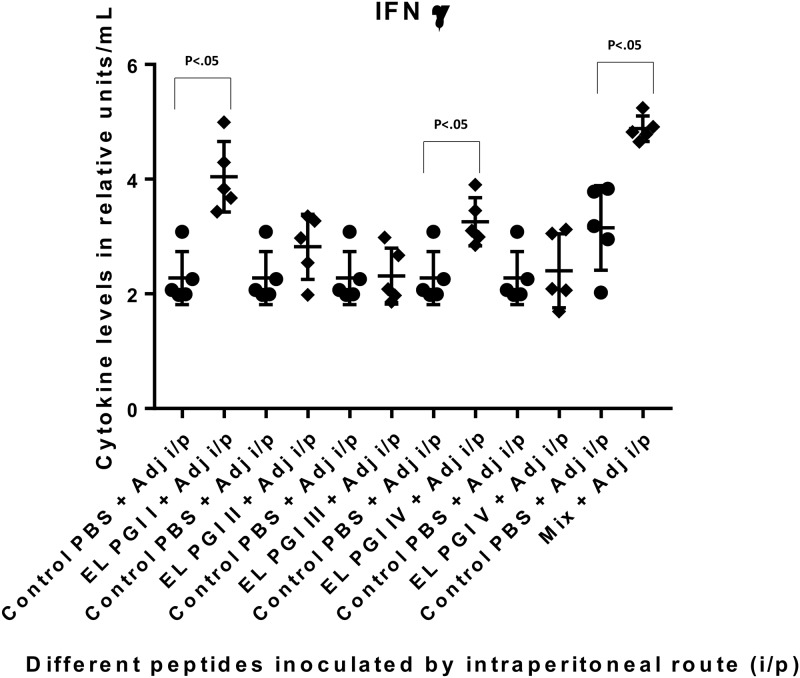
IFN-γ levels in BALB/c mice against all peptides and their mixture inoculated by intraperitoneal route. On the X-axis are shown all five peptides with the adjuvant, their mixture and controls (PBS + Adjuvant) inoculated intraperitoneally. Mice were primed with peptide + CFA and two booster doses at two weeks interval each were given with peptide + IFA. IFN-γ levels in pg/ml in the undiluted sera are shown on Y-axis. Data collected from 6 mice per group (2 separate experiments) are expressed as geometric mean concentration (± 2SD). Statistical significance was determined by Mann-Whitney U test and the p-value of < 0.05 was taken as significant.

**Fig 11:**
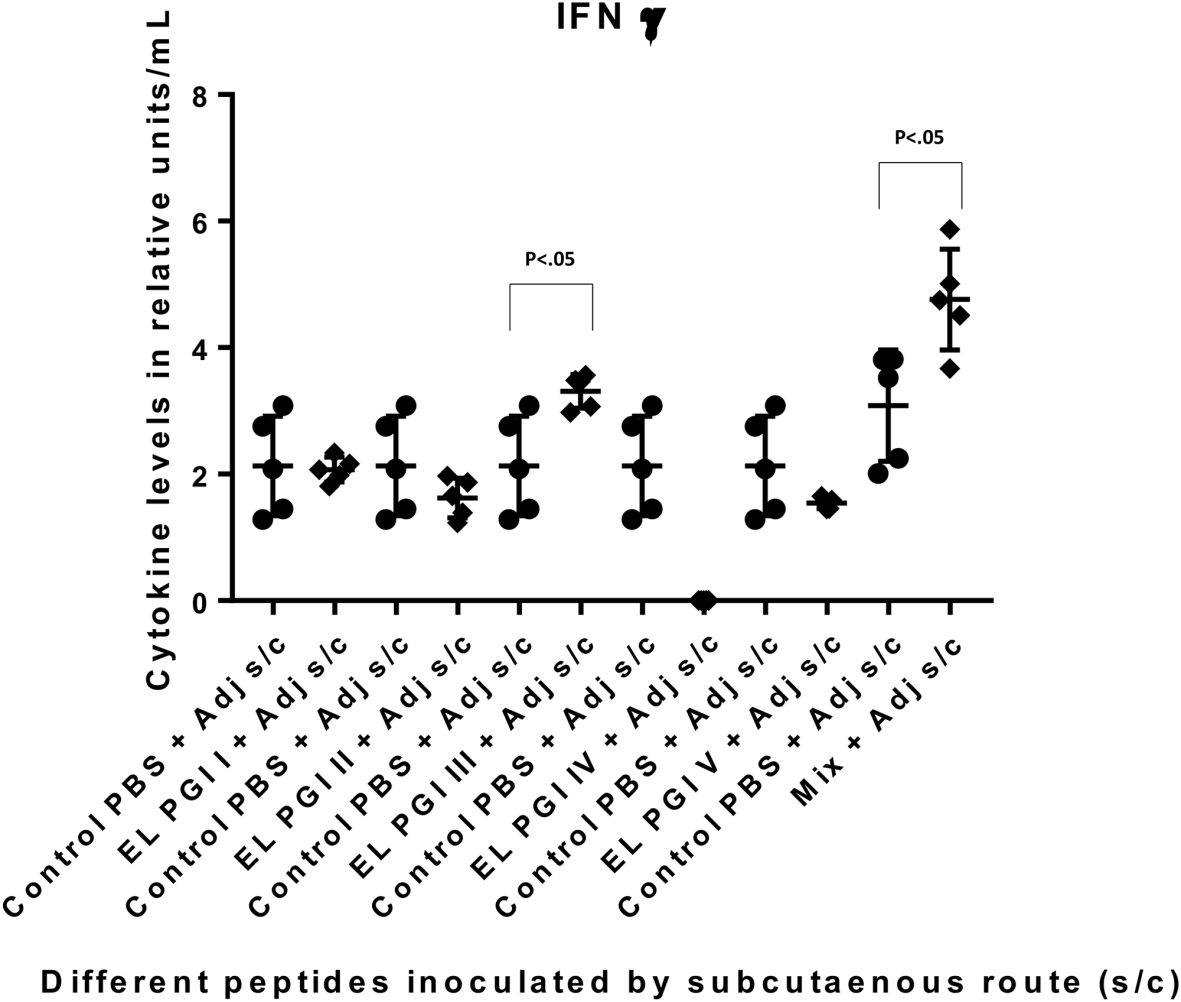
IFN-γ levels in BALB/c mice against all peptides and their mixture inoculated by subcutaneous route. On the X-axis are shown all five peptides with the adjuvant, their mixture and controls (PBS + Adjuvant) inoculated subcutaneously. Mice were primed with peptide + CFA and two booster doses at two weeks interval each were given with peptide + IFA. IFN-γ levels in pg/ml in the undiluted sera are shown on Y-axis. Data collected from 6 mice per group (2 separate experiments) are expressed as geometric mean concentration (± 2SD). Statistical significance was determined by Mann-Whitney U test and the p-value of < 0.05 was taken as significant.

**Fig 12:**
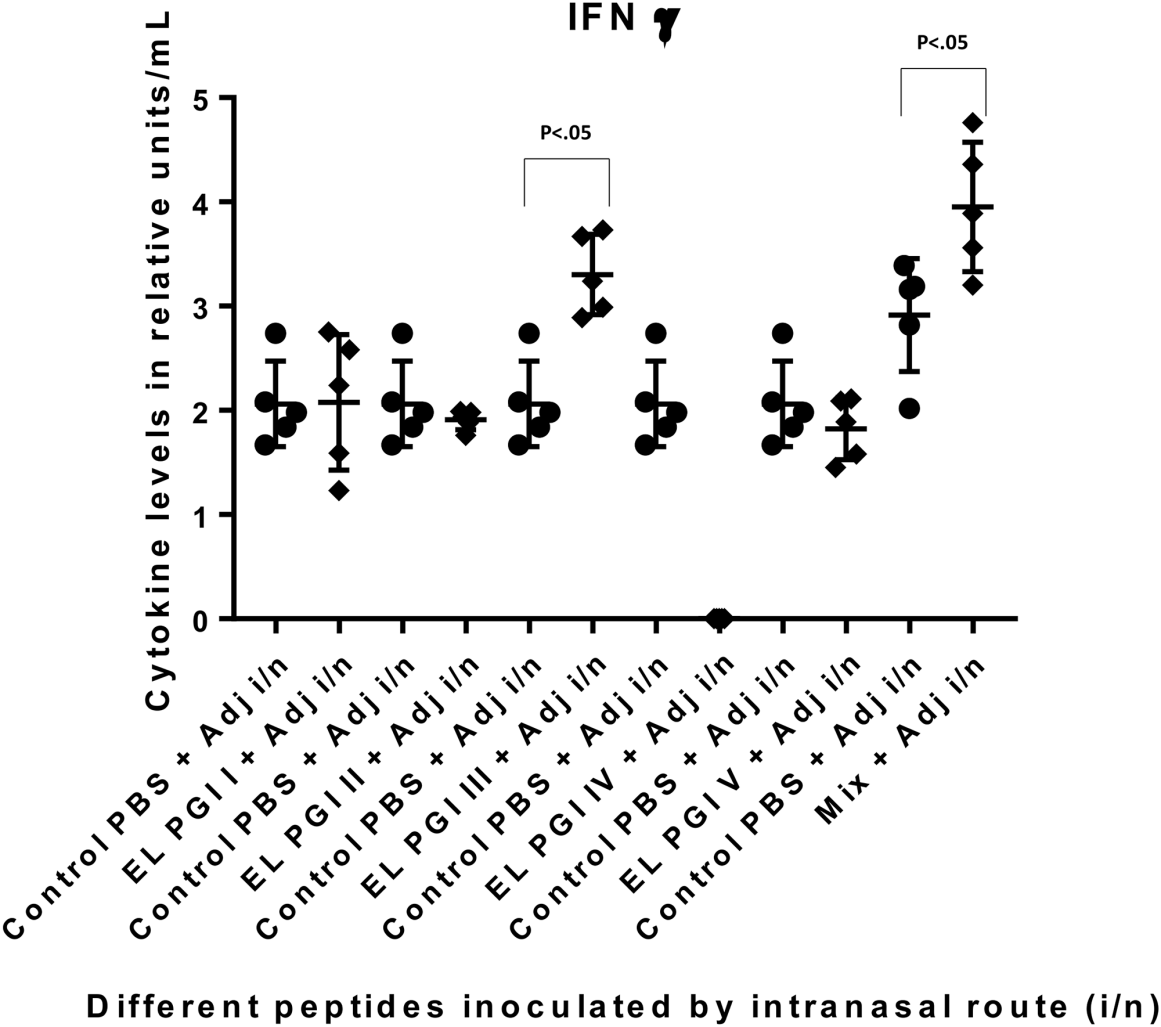
IFN-γ levels in BALB/c mice against all peptides and their mixture inoculated by intranasal route. On the X-axis are shown all five peptides with the adjuvant, their mixture and controls (PBS + Adjuvant) for each peptide inoculated intranasally. Mice were primed with peptide + CFA and two booster doses at two weeks interval each were given with peptide + IFA. IFN-γ levels in pg/ml in the undiluted sera are shown on Y-axis. Data collected from 6 mice per group (2 separate experiments) are expressed as geometric mean concentration (± 2SD). Statistical significance was determined by Mann-Whitney U test and the p-value of < 0.05 was taken as significant.

The IFN-γ level was significantly elevated against the putative lipoprotein (EL PGI I) and IcsB (EL PGI IV) by intraperitoneal route (Fig 10). Spa32 (EL PGI III) elicited significant IFN-γ by subcutaneous and intranasal routes (Figs 11 and 12). The putative heat shock protein (EL PGI II) and the putative hypothetical protein (EL PGI V) did not produce IFN-γ by any of the routes (Figs 10–12). The cocktail of all the five peptides elicited significant levels of IFN-γ by all three routes (Figs 10–12).

### 3.6 Cytokine levels in patient sera

Both Th1 and Th2 cytokines were found to be elevated in patients’ sera as compared to the healthy controls. The p-values were significant (<0.05) for IL-1β, IL-10, IFN-γ and TNF-α, but IL-2 and IL-4 levels were insignificant in shigellosis patients’ sera. The levels were checked for a comparison of cytokines profile between mice and humans. In mice TNF-α and IFN-γ were elevated. The details are attached in supplementary material (S2 Fig).

## Discussion

In the present study, we used bioinformatics approach to identify antigens conserved across major serotypes of *Shigella*. Many widely recommended prediction algorithms with high accuracy (up to 91.4%) for prokaryotes were used. We identified 48 novel immunogenic antigens by in-silico analysis and further evaluated the best five in wet-lab experiments.

The identified putative heat shock protein (HSP) elicited significant IgG and IgA antibody response by all the three routes tested and up-regulated TNF-α cytokine by intraperitoneal route. The HSPs are a diverse family of proteins having varied functions. Some have served as excellent vaccine candidates for pathogens such as *Helicobacter pylori* and *Brugia malayi* [68,69]. The second antigen, a putative hypothetical protein not only elicited IgG and IgA antibodies by subcutaneous and intranasal routes but also upregulated TNF-α cytokine by all the three routes. The IgG and IgA antibodies against above two antigens were significantly present in shigellosis patient sera.

Other selected peptides, Spa32and IcsB elicited significant IgA titers in mice. Spa32 upregulated IFN-γ and TNF-α levels by subcutaneous and intranasal routes in mice but antibody response (both IgG and IgA) against this antigen was insignificant in the patient sera, whereas IcsB had significant IgG response against it in the patient sera. The Spa32 locus consists of many proteins, which constitute the *Shigella* type III secretory system essential for invasion and spread of bacterium [70]. Studies have shown that folding defects in Spa32 interfere with Ipa secretions into inter-epithelial protrusions, thereby inhibiting cell to cell spread and thus demonstrating its role in pathogenesis and as potential vaccine candidate [71].

The fifth peptide used in the study, a putative lipoprotein did not elicit humoral immune response by any of the routes, which demonstrates that bioinformatics prediction may not be 100% accurate or in-silico and in-vivo results may differ. Lipoproteins play critical roles in nutrient uptake, antibiotic resistance, and adhesion in various pathogens [72]. However, it elicited TNF-α (by all routes) and IFN-γ by the intraperitoneal route in mice and IgG antibodies against it were significantly present in human sera. A cocktail of all five peptides was found to be more effective than the individual peptide in eliciting both humoral (IgG and IgA) and cellular immune response (TNF- α and IFN-γ). This may be due to the presence of all the predicted B-and T-cell epitopes in the mixture and these results are in concordance with a study by Hackbarth *et al* in *Pseudomonas aeruginosa* [73]. Therefore, these five peptides can be utilized in a multi-epitope vaccine.

To find the best possible route of delivery for these antigens, we inoculated them by three different routes. Subcutaneous and intranasal routes were found to be more immunogenic than intraperitoneal route. In the subcutaneous route, antigen is released slowly to antigen presenting cells (APCs) as compared to intraperitoneal route [74]. The intranasal route on the other hand has an added advantage of eliciting the immune response at local as well as distant sites, contributing to gut immune response along with systemic immune response [75]. Therefore, either subcutaneous or intranasal route can be useful in the peptide delivery.

The data from human infections and experiments in animals have shown that both systemic and mucosal arms of humoral immunity are activated during shigellosis [76,77]. Shigellae are locally invasive pathogens and induction of mucosal immunity should be highly desirable but the correlates of protective immunity are controversial in the case of shigellosis. It is still a contentious issue whether mucosal IgA antibodies or serum IgG antibodies are protective against *Shigella* [78]. Some studies have reported strong mucosal sIgA antibody response following natural infection/experimental challenge, whereas in some, serum anti-O-antigen IgG antibodies were reported protective [25,79–81]. Moreover, many studies have demonstrated that systemic immunization is capable of inducing the immune response at mucosal sites, as exemplified in the cases of polio and influenza viruses [82–84]. Therefore, keeping in view the above findings, we chose CFA as adjuvant because of its wide spread use in the peptide immunization for increasing humoral and cellular immunity [85–87]. For peptide formulations in humans, the adjuvant like DMT-liposome can be highly useful, which is a cocktail of dimethyl di-octa-decyl ammonium bromide (DDA), monophosphoryl lipid-A (MPL) and trehalose dicoryno-mycolate (TDM) as it has been successfully tested for tuberculosis subunit vaccine [88]. We have also reported the cellular immune response in this study. TNF-α and IFN-γ cytokines were elevated in mice sera, which reveals a strong Th1 mediated immune response in mice. In some studies, only Th1 mediated response was observed, whereas others have shown both Th1 and Th2 mediated response [89]. In this study, shigellosis patient sera exhibited elevated cytokines levels for IL-1β, IL-10, TNF-α and IFN-γ and our findings are similar to that of Raqib et al [90]. The difference in the pattern of cytokine elevation in mice vs. humans in our study may be explained based on host adaptation. Shigellae are highly host-adapted and the immune response in mice may be different from humans but in both mice as well as humans, the up-regulation of TNF-α and IFN-γ has shown that these two cytokines play a crucial role in immunity [91,92].

We further investigated the functions of the five selected proteins by widely used software, InterProScan (https://www.ebi.ac.uk/interpro/sequence-search). Putative lipoprotein was identified as a bacterial protein, absent in primates and its function is not clear. Putative heat shock protein was found to be involved in the proteolysis and metallo-endopeptidase activity. Spa32 was identified as a member of the type III secretory pathway and thus involved in virulence activity. IcsB was found to be linked to the virulence mechanism. Hypothetical protein identified as a bacterial protein, belongs to MltA-interacting protein super-family involved in the peptidoglycan remodelling. The above findings confirm the roles of the selected proteins in the pathogenesis of shigellosis. We further checked the pathogenicity of these proteins by MP3 software (Predict Pathogenic Proteins in Metagenomes) (metagenomics.iiserb.ac.in/mp3) [93]. Four of the tested proteins, except the lipoprotein (EL PGI I), were predicted to be pathogenic. These findings have increased their probability to be tested as vaccine candidates for *Shigella*.

This study demonstrates a new way of identifying antigens that can serve as vaccine candidates. The antigens reported in this study are unique and have not been tested so far against *Shigella* and antibodies against these are present in shigellosis patients’ sera. These antigens can be an alternative to serotype-specific antigens used for vaccines in the past. These antigens are cross protective and conserved over major serotypes of *Shigella*, therefore can be used in global vaccine development against *Shigella*. The presence of natural antibodies against these antigens in Shigellosis patients’ sera further supports their use as future vaccine candidates. In future, animal challenge studies are required to establish the protective efficacy of these immunogens.

## Supporting information

S1 Fig List of outer membrane or secreted proteins after localization predictions. The excel sheet contains the common proteins identified after BLAST and subjected to localization predictions by various softwares. The file contains proteins predicted as outer membrane or secreted proteins.
(XLSX)

S2 Fig Different cytokines expression in shigellosis patients’ versus healthy controls sera. The graph shows different cytokines levels expressed in pg/ml in the sera of patient versus controls. The statistical significance was calculated by Independent t-test and p-values < 0.05 were taken as significant.
(TIF)

S3 Fig Peptide sequences after B- and T-cell epitopes predictions. The file shows around 39,000 peptides common after B- and T-cell epitope predictions. Out of these, 48 were of required length (≥9 amino acids) and best five from these were selected for in-vivo work.
(XLSX)
