# Supplementary figures and images for: In silico analysis to identify vaccine candidates common to multiple serotypes of Shigella and evaluation of their immunogenicity
Source: PLoS One. 2017 Aug 2;12(8):e0180505. doi: 10.1371/journal.pone.0180505 (PMC5540609; doi:10.1371/journal.pone.0180505)

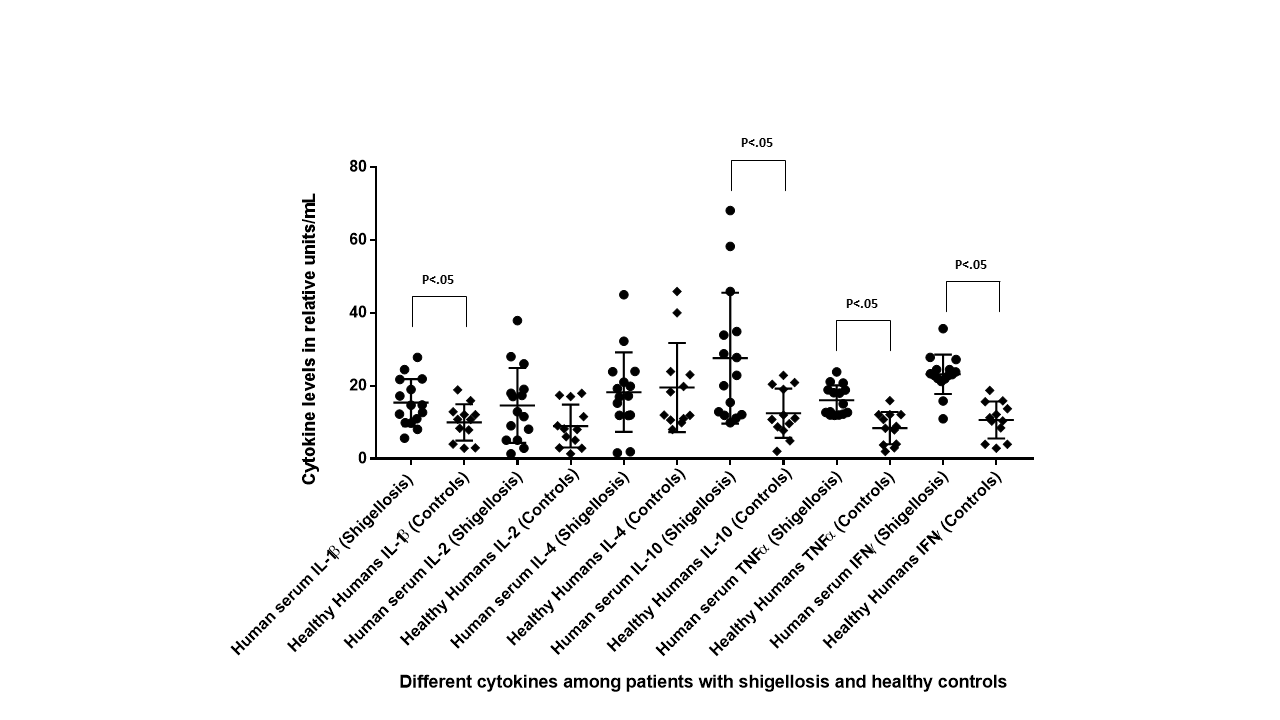

Supplement: S2 Fig — The graph shows different cytokines levels expressed in pg/ml in the sera of patient versus controls. The statistical significance was calculated by Independent t-test and p-values < 0.05 were taken as significant. (TIF) [file pone.0180505.s002.tif]
